# Cross-Validation of a Multiplex LC-MS/MS Method for Assaying mAbs Plasma Levels in Patients with Cancer: A GPCO-UNICANCER Study

**DOI:** 10.3390/ph14080796

**Published:** 2021-08-12

**Authors:** Clémence Marin, Nihel Khoudour, Aurélien Millet, Dorothée Lebert, Pauline Bros, Fabienne Thomas, David Ternant, Bruno Lacarelle, Jérôme Guitton, Joseph Ciccolini, Benoit Blanchet

**Affiliations:** 1 SMARTc, CRCM INSERM U1068, Aix-Marseille Universiteé, F-13009 Marseille, France; clemence.marin93@gmail.com (C.M.); bruno.lacarelle@ap-hm.fr (B.L.); ciccolini.joseph@gmail.com (J.C.); 2 Laboratoire de Pharmacocinétique et Toxicologie, La Timone University Hospital of Marseille, F-13385 Marseille, France; 3 COMPO, CRCM INSERM U1068-Inria, Aix-Marseille Universiteé, F-13385 Marseille, France; 4 Department of Pharmacokinetics and Pharmacochemistry, Cochin Hospital, AP-HP, CARPEM, F-75014 Paris, France; nihel.khoudour@aphp.fr; 5 Laboratory of Biochemistry and Pharmacology-Toxicology, Centre Hospitalier Lyon-Sud, Hospices Civils de Lyon, F-69495 Pierre Bénite, France; aurelien.millet@chu-lyon.fr (A.M.); Jerome.guitton@univ-lyon1.fr (J.G.); 6 Promise Proteomics, 7 Parvis Louis Néel, F-38040 Grenoble, France; dorothee.lebert@promise-proteomics.com (D.L.); pauline.bros@promise-proteomics.com (P.B.); 7 Institute Claude Regaud, Institut Universitaire du Cancer (IUCT)–Oncopole, F-31059 Toulouse, France; Thomas.Fabienne@iuct-oncopole.fr; 8 Centre de Recherches en Cancérologie de Toulouse (CRCT), INSERM UMR1037, University Paul Sabatier, Toulouse III, F-31037 Toulouse, France; 9 EA 4245 “Transplantation, Immunology, Inflammation”, Department of Clinical Pharmacology, University of Tours, F-37032 Tours, France; david.ternant@univ-tours.fr; 10 CHRU of Tours, F-37200 Tours, France; 11 Department of Toxicology, Faculty of Pharmacy, University Lyon 1, F-69373 Lyon, France; 12 UMR8038 CNRS, U1268 INSERM, Faculty of Pharmacy, University of Paris, PRES Sorbonne Paris Cité, CARPEM, F-75006 Paris, France

**Keywords:** LC–MS/MS, biologics, therapeutic drug monitoring, cross-validation, immunotherapy

## Abstract

Background: Different liquid chromatography tandem mass spectrometry (LC–MS/MS) methods have been published for quantification of monoclonal antibodies (mAbs) in plasma but thus far none allowed the simultaneous quantification of several mAbs, including immune checkpoint inhibitors. We developed and validated an original multiplex LC–MS/MS method using a ready-to-use kit to simultaneously assay 7 mAbs (i.e., bevacizumab, cetuximab, ipilimumab, nivolumab, pembrolizumab, rituximab and trastuzumab) in plasma. This method was next cross-validated with respective reference methods (ELISA or LC–MS/MS). Methods: The mAbXmise kit was used for mAb extraction and full-length stable-isotope-labeled antibodies as internal standards. The LC–MS/MS method was fully validated following current EMA guidelines. Each cross validation between reference methods and ours included 16–28 plasma samples from cancer patients. Results: The method was linear from 2 to 100 µg/mL for all mAbs. Inter- and intra-assay precision was <14.6% and accuracy was 90.1–111.1%. The mean absolute bias of measured concentrations between multiplex and reference methods was 10.6% (range 3.0–19.9%). Conclusions: We developed and cross-validated a simple, accurate and precise method that allows the assay of up to 7 mAbs. Furthermore, the present method is the first to offer a simultaneous quantification of three immune checkpoint inhibitors likely to be associated in patients.

## 1. Introduction

Currently more than 25 monoclonal antibodies (mAbs) have been approved for treating cancer by the US Food and Drug Administration (FDA), and European Medicines Agency (EMA). Usually, “classical” monoclonal antibodies (e.g., bevacizumab, cetuximab, rituximab, trastuzumab) are distinguished from immune checkpoint inhibitors (e.g., ipilimumab, nivolumab, pembrolizumab, atezolizumab) because of their mechanism of action. mAbs usually target circulating or membrane antigens involved in tumor proliferation such as EGFR (epithelial growth factor receptor), VEGF (vascular endothelial growth factor), CD20 or HER–2 receptor. Over the last decade, the use of mAbs able to modulate anti-tumor immune response has been spreading. These immunotherapies such as checkpoint inhibitors are directed against targets involved in silencing anti-tumoral immune response like PD-1 (programmed cell death receptor), PD-L1 (programmed death-ligand 1), or CTLA4 (cytotoxic T-lymphocyte antigen-4). As such, they do not interfere directly with proliferation and differentiation of cancer cells, but rather aim at harnessing tumor immunity to trigger some kind of immune-related cell death. Although those mAbs have usually proven clinical benefit with acceptable safety in daily clinical practice in paradigmatic settings such as lung cancer, melanoma, head and neck or renal cancer, the variability in the clinical outcomes remains largely unpredictable. In the context of personalized medicine, the determinants of this clinical variability should be identified to optimize response or to propose other treatment modalities.

The inter-individual variability observed in clinical response could be, at least in part, attributed to the pharmacokinetics variability of mAbs. Indeed, exposure levels or more rarely, pharmacokinetic parameters, such as total clearance have already been associated with pharmacodynamic endpoints (i.e., overall and progression-free survival, efficacy) for bevacizumab [1], rituximab [2,3] and cetuximab [4,5]. For instance, 34 µg/mL plasma level threshold for efficacy was proposed for cetuximab in head and neck cancer [4] and 15.5 µg/mL for bevacizumab in metastatic colorectal cancer [1]. Given the large interindividual pharmacokinetic variability reported with most biologics [6,7], predicting whether a patient will be adequately exposed to ensure maximal target engagement can be tricky. Regarding immunotherapies, data are less convincing thus far. It has been clearly documented that exposure–response relationships exist for anti-CTLA4 ipilimumab [8] as shown both in phase II [9,10] and phase III studies [11]. By contrast, pharmacokinetic/pharmacodynamics (PK/PD) data about nivolumab and pembrolizumab are more contradictory. Most studies reported that the PK/PD relationships are flat for both mAbs, whereas a single study evidenced an exposure efficacy with nivolumab [12,13,14]. Some authors argue that PK/PD relationships do exist with immune checkpoint inhibitors, but with respect to the extremely high dosing approved for those drugs, usually plasma levels largely exceed the threshold concentration required to ensure a maximal target engagement [15]. However, to what extent those theoretical large amounts of mAbs are sufficient to ensure proper target engagement despite the PK variability remains unclear. In addition to possible impact on drug efficacy, several groups have suggested that patients treated with immune checkpoint inhibitors could be overdosed with respect to the efficacy thresholds. This calls for developing therapeutic drug monitoring (TDM) to possibly customize the frequency of the administration, i.e., by adapting the scheduling to the decay in plasma levels [16].

Regardless of the context, plasma monitoring of mAbs could be a useful tool for clinical decision making. To achieve TDM with biologics, robust, specific, and validated bioanalytical methods are required. As of today, most of the methods for mAbs quantification in plasma such as phase I studies are based upon ELISA methods [5,17,18,19,20,21,22,23] which do not necessarily meet the time- and cost-effectiveness requirements of routine drug monitoring, especially in real-world patients. In addition, the limitations in terms of specificity have led to the development of alternative analytical strategies that should be therefore both time- and cost-effective. Finally, ELISA methods are not easily adaptable for multiplexed assays. Recently, some multiplex assays based on liquid chromatography coupled with tandem mass spectrometry (LC–MS/MS) were proposed [23,24,25]. Most of these methods can simultaneously quantify several biologics in a single run, but thus far none allowed the simultaneous quantification of several immune checkpoint inhibitors. The increasing number of mAbs used in monotherapy or in combination with other mAbs support the need of multiplex assays for mAbs, to meet the time- and cost-effectiveness requirements of routine TDM. 

Here, we developed and validated a multiplexed LC–MS/MS method using a ready-to-use kit for the simultaneous plasma quantification of up to 7 mAbs frequently used in oncology (i.e., bevacizumab, cetuximab, nivolumab, ipilimumab, pembrolizumab, rituximab, and trastuzumab), over a large range of concentrations in order to be used for drug monitoring as well as PK/PD studies. Then, this method was cross-validated with published reference methods (i.e., ELISA or LC–MS/MS).

## 2. Results

### 2.1. Validation

#### 2.1.1. Chromatograms

**Figure 1:**
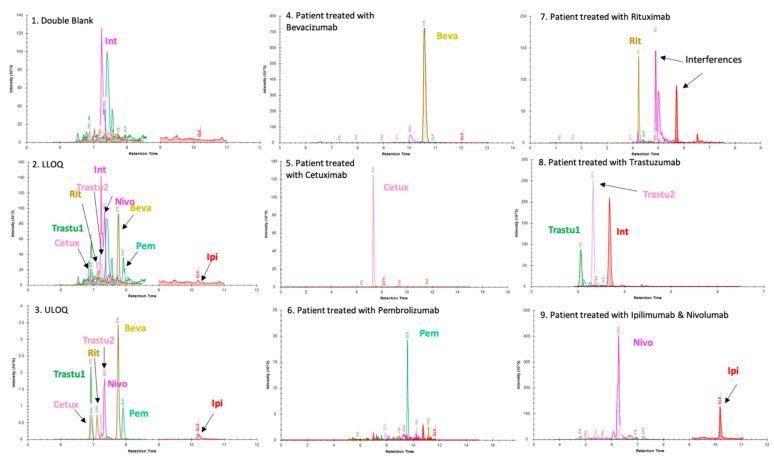
Panels (**1**), (**2**) and (**3**) present chromatographic profiles of double blank drug-free plasma matrix, lower limit of quantification (LLOQ) and upper limit of quantification (ULOQ), respectively. Int: interference. These chromatograms were obtained using a BioZen™ 2.6 µm Peptide XB–C18 LC column and the LC–MS parameters were those described in the Materials and Methods section. Two proteotypic peptides were selected for trastuzumab because both are unique and give intense signals in LC–MS. For the other mAbs, the list of proteotypic peptides was limited because of the sequence homology of mAbs with endogenous IgG (other peptides are not specific). In this context, a single peptide was selected in the quantification method of all mAbs except trastuzumab. Panels (**4**), (**5**), (**6**), (**7**), (**8**) and (**9**) display chromatograms obtained from plasma samples of patients treated with bevacizumab (Beva) (**4**), cetuximab (Cetux) (**5**), pembrolizumab (Pem) (**6**), rituximab (Rit) (**7**), trastuzumab (Trastu1 & Trastu 2) (**8**) and combination therapy nivolumab (Nivo) + ipilimumab (Ipi) (**9**). The retention times of mAbs shown in these panels are different with those observed in panels (**1**), (**2**) and (**3**) because of the use of different chromatographic systems and columns in each laboratory using the kit. Thus, panels (**1**), (**2**), (**3**) were analyzed on SCIEX 6500QTRAP by Promise Proteomics. Panels (**4**) and (**9**) were analyzed on SCIEX 6500 QTRAP by PROMISE Proteomics with distinct LC parameters. Panels (**5**), (**6**), (**7**) and (**8**) were analyzed on a THERMO TSQ Altis at Cochin hospital (Paris, France). Importantly and as visible on (**1**), (**2**) and (**3**), an interfering signal (Int) is present for nivolumab peptide when analyzing plasma samples with a QQQ mass spectrometer. It elutes very close to the peak of interest. This interference can be separated with an appropriate gradient. Lower limit of quantification and linearity.

Figure 1 presents typical chromatographic profiles.

**Figure 2:**
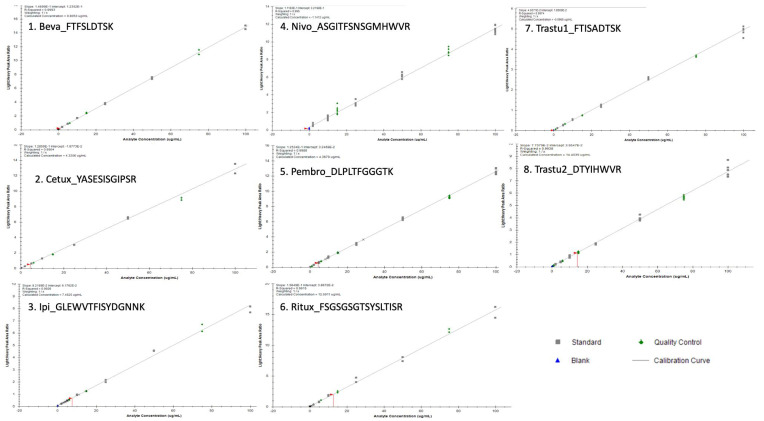
Slopes, intercepts and coefficient of determination mean values were respectively as follows (*n* = 6): y = 0.15X + 0.025 (*r*^2^ = 0.999) for bevacizumab (Beva), y = 0.14X + 0.012 (*r*^2^ = 0.999) for cetuximab (Cetux), y = 0.052X + 0.26 and (*r*^2^ = 0.986) for Ipi, y = 0.11X + 0.32 (*r*^2^ = 0.995) for Nivo, y = 0.13X + 0.032 (*r*^2^ = 0.998) for pembrolizumab (Pembro), y = 0.16X + 0.044 (*r*^2^ = 0.996) for rituximab (Ritux), y = 0.05X + 0.018 (*r*^2^ = 0.997) for trastuzumab_pep1 (Trastu1) and y = 0.08X + 0.040 (*r*^2^ = 0.994) for trastuzumab_pep2 (Trastu2) where x is the concentration in µg/mL and y is the area ratio. Selectivity, carry-over and matrix effect.

For all mAbs, the LLOQ was 2 µg/mL corresponding to the lowest level of calibration. Standards curves were linear from 2 to 100 µg/mL for all mAbs. Over this concentration range, the regression coefficient (*r*^2^) of the calibration curves was always greater than 0.994 with back-calculated calibration samples within ±15% (±20% at LLOQ), as shown in Figure 2. Signal to noise (S/N) ratios calculated by dividing signal area at LLOQ by signal area in double blank samples were 33, 41, 11, 8, 25, 10, 30 and 8 for Beva, Cetux, Ipi, Nivo, Pembro, Ritux, and Trastu-1 or Trastu-2 peptides, respectively. All the validation criteria for linearity were fulfilled.

**Figure 3:**
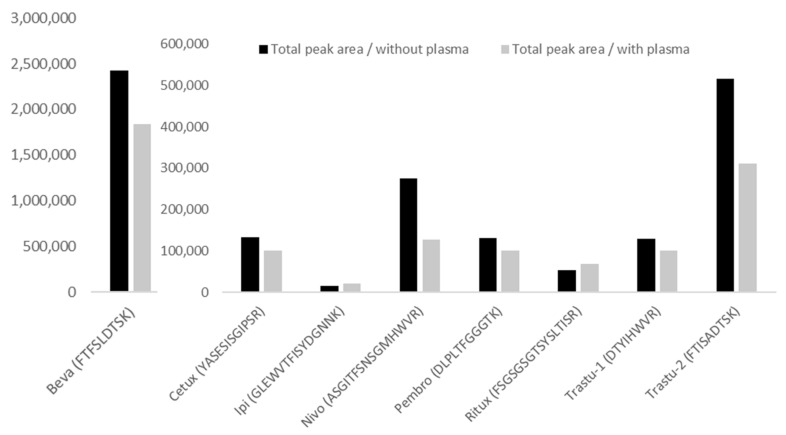
Matrix effect for the different mAbs. Black histograms represent the total area measured for the peptide (mean of MRM transitions) in the absence of plasma, while the grey histograms represent the total area measured for the same peptide and same concentration (mean of MRM transitions) in the presence of plasma. Samples, without and with plasma, were analyzed in 6 replicates from a pool of human plasma samples. CV% between replicates were consistent and below 9% in the absence of matrix, and below 11% in the presence of matrix, except for Nivo where CV% was 19% in the presence of matrix.

No interference was observed at the retention times of analytes and IS in blank samples extracted from the five tested human samples. Regarding Nivo peptide detection (i.e., peptide ASGI), based on our experience, an interference can be observed in most of the patient samples. It was crucial to correctly separate this interference, using adequate LC parameters. Regarding matrix effect (Figure 3), the mean for the 7 mAbs was −12.7% (CV = 35.5%). The lowest value was −54% for Nivo and the highest of +33% for Ipi. 

The matrix effects measured are −24%, −25%, +33%, −54%, −23.5%, +25.8%, −39.7% and −22.1% for Beva, Cetux, Ipi, Nivo, Pembro, Ritux, Trastu-1 and Trastu-2 peptides, respectively.

#### 2.1.2. Accuracy and Precision

Table 1 presents the results of within-day and between-day precision (expressed as coefficient of variation, CV) and accuracy. Inter-assay precision and accuracy for all mAbs ranged from 1.0–13.1% and 91.3–107.1%, respectively. Intra-assay precision and accuracy were <14.6% and ranged from 90.1 to 111.1%, respectively. Bias and CV did not exceed 20% for LLOQ and 15% for three levels of internal quality control (IQC) for at least 1 peptide, therefore the acceptance criteria for accuracy and precision were therefore met for all mAbs. In addition, we evaluated the performance of the method with one of Trastu biosimilar (Ontruzant^®^). The accuracy and precision for 3 IQCs (*n* = 6 for each) ranged from 99.8 to 110.4% and from 2.1 to 8.9%, respectively (Table S1).

#### 2.1.3. Dilution Effect

The accuracy and precision of 5-fold diluted plasma sample (*n* = 6 for each mAb) ranged from 92.4 to 106.8% and from 1.4 to 9.3%, respectively (Table S2).

### 2.2. Comparison of mAbs Levels with LC–MS/MS Method versus References Methods

**Figure 4:**
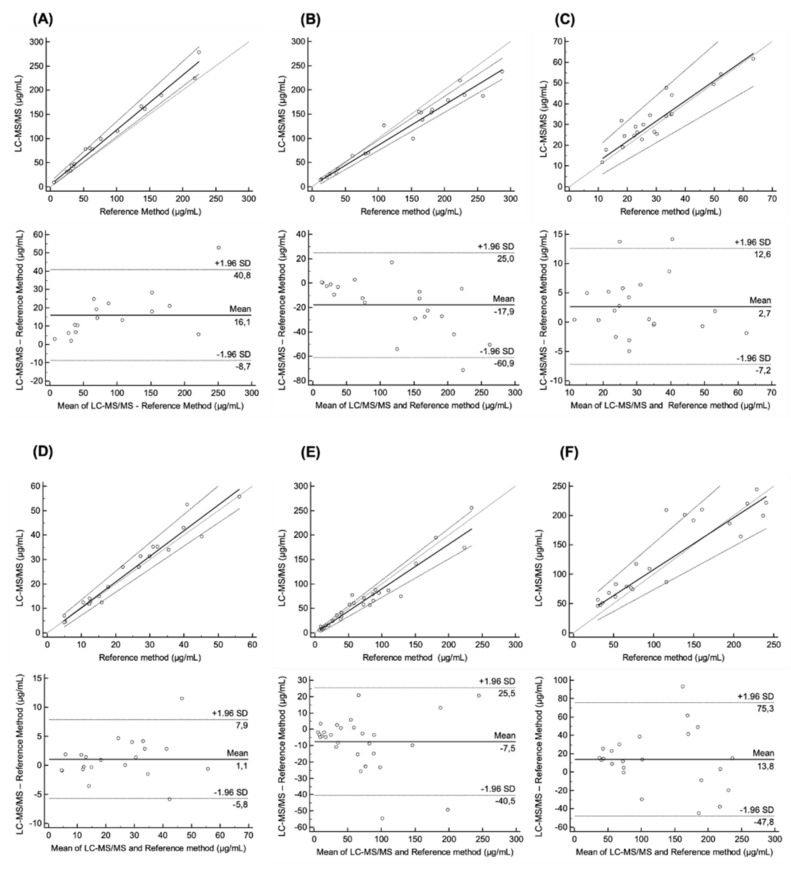
Passing-Bablok and Bland–Altman plots: Passing-Bablok regression plot of concentrations measured by LC–MS/MS and reference method for (**A**) bevacizumab (*n* = 16), (**B**) cetuximab (*n* = 21), (**C**) nivolumab (*n* = 21), (**D**) pembrolizumab (*n* = 21), (**E**) rituximab (*n* = 28), (**F**) trastuzumab (*n* = 23) in patients with advanced cancers. Bland–Altman analysis of the difference between LC–MS/MS and reference method for (**A**) bevacizumab, (**B**) cetuximab, (**C**) nivolumab, (**D**) pembrolizumab, (**E**) rituximab, (**F**) trastuzumab. The mean ± 1.96 standard deviation lines (95% limits of agreement) are plotted for reference.

A total of 142 plasma samples were assayed. The median concentration (range) was 88.5 (6.1–225.2) μg/mL for Beva (*n* = 16), 152.7 (13.2–288.0) μg/mL for Cetux (*n* = 21), 3.9 (1.1–14.2) μg/mL for Ipi (*n* = 12), 28.0 (11.4–63.5) μg/mL for Nivo (*n* = 21), 26.8 (4.2–55.5) μg/mL for Pembro (*n* = 21), 57.7 (7.4–234.9) μg/mL for Ritux (*n* = 28) and 95.4 (30.4–241.0) μg/mL for Trastu (*n* = 23). The interchangeability of the present multiplex LC–MS/MS method could not be tested for Ipi due to the lack of international laboratories able to assay this mAb. Overall, 130 plasma samples were analyzed for the cross-validation. Figure 4 presents Passing Bablok and Bland–Altman plots for each assay pair.

The Passing-Bablok regression revealed no significant deviation from linearity for all mAbs (Cusum test). In comparison with reference methods, the multiplex LC–MS/MS method overestimated concentrations by 13.2% for Beva and 4.9% for Pembro, while it underestimated concentrations for Cetux, Nivo, Ritux and Trastu (17.1%, 3.0%, 9.1%, and 11.6%, respectively). Table 2 summarizes the method agreement between each assay pair. Results showed that the mean absolute bias of measured concentrations between multiplex and reference methods was 9.82% (range 3.0–17.1%). Overall, these results suggest the interchangeability of the present multiplex LC-MS/MS method with published reference methods for Beva, Cetux, Nivo, Pembro, Ritux and Trastu.

## 3. Discussion

Over the last decade, the literature about pharmacokinetics and pharmacodynamics of mAbs used in oncology has significantly expanded [32,33,34]. A large inter-individual variability in pharmacokinetics parameters and subsequent exposure levels is usually reported, regardless of the type of mAbs considered. By contrast, inconsistencies were observed when reporting on PK/PD relationships with mAbs. For instance, trough levels or clearance values were repeatedly associated with the efficacy of anti-EGFR cetuximab either in colorectal or head and neck cancers [4,5,35,36]. Similar exposure–effects relationships were found with anti-VEGF bevacizumab [1] or anti-HER2 trastuzumab [19]. With immune checkpoint inhibitors, both efficacy and toxicity endpoints seemed to be associated with plasma concentrations of anti-CTLA4 ipilumumab [9]. Oppositely, contradictory findings were published with anti-PD1 nivolumab in lung cancer patients [35,36] and flat relationships were suggested with anti-PD1 pembrolizumab [37]. Overall, the very existence of PK/PD relationships with mAbs remains a controversial issue in clinical oncology. Several explanations can help in understanding those erratic findings. First, clearance of mAbs can be influenced by target-mediated drug disposition (TMDD), a phenomenon making PD endpoints such as tumor burden a relevant covariate for predicting clearance values of mAbs, the higher the antigenic mass, the higher the clearance and the lower the drug plasma levels [38]. Therefore, whether low concentrations of mAbs are the cause or the consequence of increase in tumor burden is tricky to understand. Of note, TMDD does not apply to anti-CTLA4 or anti-PD1 mAbs since target engagement is not related to the tumor burden but rather to the immune system. Another possible confounding factor is the fact that low albumin levels (i.e., cachexia frequently observed in patients with progressive disease) is another factor likely to increase mAbs clearance [35], thus further blurring the picture when trying to understand whether PK is the cause or the consequence of disease evolution. In addition, to better understand PK/PD relationships, the fact that most, if not all, immune checkpoint inhibitors administered now as flat doses could yield plasma levels largely exceeding the threshold required for target engagement [16,39,40] calls for developing tools for evaluating exposure to mAbs. For example, TDM of immune checkpoint inhibitors (ICIs), could be interesting, not necessarily as an attempt to tailor dosing to increase efficacy, but at least to customize the frequency of administrations, in a drug cost saving perspective [16]. Indeed, TDM-based determination of individual PK parameters could allow simulating the time to reach the efficacy threshold, and to determine when the next dose should be administered for a given patient [41]. A new bioanalytical method for TDM application in routine should meet different analytical requirements such as sensitivity, precision, and accuracy, in addition to ease of use, cost and time effectiveness considerations. Furthermore, the increasing use of combination therapy with multiple mAbs calls for multiplex assays. As far as we know, this is the first report of a validated LC–MS/MS method able to simultaneously assay up to 7 therapeutic mAbs including three check point inhibitors (i.e., Ipilimumab, Nivolumab and Pembrolizumab). Co-administration of ICIs such as Ipi plus Nivo or Pembro has become a common practice. To date, only 10 bioanalytical methods [18,20,28,39,40,42,43,44,45,46] including 5 LC–MS/MS methods [28,39,40,43,44] have been published for these 3 mAbs, but none proposes a simultaneous assay as ours. The present method could become the bioanalytical method of choice to explore PK/PD relationship of ICIs, especially in patients treated in combination.

According to the EMA guidelines for bioanalytical method evaluation [47], the present multiplex LC–MS/MS method meets all the current validation criteria, except for matrix effect evaluation. Different MRM transitions used as “quantifier” gave consistent quantification data, regardless of the mAb. In this context, we preferred calculating the mAb concentration by averaging the signal of multiple quantifiers to gain sensitivity and reliability. The validation showed satisfying intra- and inter-day accuracy (90.1–111.1%) and precision (<14.6%) for all mAbs. Regarding Nivo quantification, an interference was observed using QQQ mass spectrometer. However, this interference would not be expected with HRMS mass spectrometer because of its greater precision in *m*/*z* measurement as compared with QQQ (~10 ppm vs ~ 0.6–0.7 Da, respectively). During the analytical validation steps, this interference was correctly separated and always eluted a few seconds before the peak of nivolumab (Figure 1, panel 1). In case of insufficient separation, the accuracy at LLOQ and low IQC for Nivo did not meet the acceptance criteria, thus impacting on low plasma concentrations (i.e., <6.0 µg/mL). This interference is probably due to a peptide from a plasma protein such as physiological IgG which has a *m*/*z* and a sequence very close to the peptide of interest when assaying Nivo. To be sure that the interference is correctly separated from Nivo, users should therefore analyze double-blank plasma sample and a blank plasma sample (i.e., matrix spiked with the stable labelled internal standard, which does not show any interference). The contaminating peak visible in the double-blank should have a different retention time than the peak of the labeled peptide visible in the blank sample. 

Based on our knowledge, mAbXmise kit is the first solution including stable-labeled mAbs and reagents and consumables for performing therapeutic mAb quantification. The use of full-length stable-isotope-labeled antibodies is an asset in comparison with most other LC–MS/MS methods which use a labeled reference peptide [4,48,49,50]. Indeed, adding a full-length stable-isotope-labeled antibody at the very beginning of extraction procedure is known to better compensate recovery and matrix effect than labeled peptides or universal stable labeled mAb [48]. In the present study, the matrix effect of all mAbs, is significant, especially for nivolumab for which a previous study already reported a high matrix effect [28]. Given the fact that the matrix effects of full-length stable-isotope-labeled antibodies were not assessed and therefore not taken into account for estimation of the matrix effect of mAbs, these results should be interpreted cautiously. However, one can expect that the use of full-length stable-isotope-labeled antibody should significantly minimize the matrix effect. Finally, the satisfying results of the cross-validation for all mAbs (except Ipi) suggests the absence of significant impact of matrix effect on the present LC–MS/MS assay.

LC–MS/MS methods decrease the inter-batch and inter-operator analytical variability as compared with canonical ELISA methods. Here, the use of ready-to-use industrial kits is a further plus ensuring better inter-batch and inter-laboratory consistency in a context of TDM or multicentric PK study. A large range of plasma concentrations (i.e., 2–100 μg/mL) was covered by the assay, including the concentrations reported during clinical PK studies [1,4,9,12,18,24,25]. Furthermore, the evaluation of dilution accuracy showed that concentrations up to 5-fold above ULOQ were adequately quantified. Our 2 μg/mL LLOQ is usually higher than those previously reported with ELISA methods [5,17,18,19,20,21,22,23], indicating a poorer sensitivity of the LC–MS/MS technique. However, with respect to the plasma concentrations usually upon mAbs administration, this LLOQ at 2 μg/mL was considered as sensitive enough in routine setting because the range of concentrations is consistent with the concentrations expected in daily clinical practice. In the present study, many samples had concentrations above the ULOQ, especially for Beva, Cetux, Ritux and Trastu. For those mAbs, blood samples were collected in patients from clinical trials. The methodology of these studies included measuring peak plasma levels, which explains that many samples were superior to ULOQ. This could be fixed by a systematic dilution of all samples withdrawn at the end of the infusion. According to a recent review of literature [6], target trough concentrations >15.5, 33.8, 25 and 20 µg/mL are proposed for Beva (colorectal cancer), Cetux (head and neck cancer), Ritux (lymphoma) and Trastu (breast cancer), respectively. Therefore, the range of plasma concentrations (2–100 μg/mL) covered by our multiplex LC–MS/MS method is fully suitable to drug monitoring in daily clinical practice. 

Finally, this multiplex LC–MS/MS method outperforms standard ELISA methods in terms of time- and cost-saving perspectives, thus fully meeting the requirements for implementing routine TDM in oncology.

The LC–MS/MS methods previously published for measuring plasma mAb levels were appropriately validated following the EMA guidelines [47]. However, very few of them were cross-validated with another method [22,24,28,30,51,52,53]. In a context of TDM, the cross-validation issue is critical for determining whether the obtained data are reliable, and whether they can be compared and used with respect to data from the literature. The present LC–MS/MS method was successfully cross-validated for all mAbs (except Ipi) as demonstrated by the consistent results between our multiplex LC–MS/MS method and reference bioanalytical methods. Indeed, the Cusum test was not statistically significant for each mAb, thus confirming the linear relationships between the methods. Furthermore, the under or overestimation of the results from this multiplex LC–MS/MS method ranged from −17.1% to 13.2%, which was satisfying. The intercept of Passing-Bablok regression for Nivo was higher than those for other mAbs. However, two PK/PD studies reported that the trough plasma concentration of nivolumab ranges from 10 to 25 µg/mL after a single infusion and 45 to 80 µg/mL at steady-state [35,36]. Consequently, this higher intercept should not have any significant consequence on drug monitoring and further decision making. Bland–Altman analysis showed that mean estimated bias for each mAb was acceptable with respect to plasma concentrations observed in patients and should have no incidence on results interpretation either. Following EMA guidelines for bioanalytical method validation [47], more than 67% of individual concentration differences must lower than 20% for each mAb when comparing bioanalytical methods. This condition was verified here. 

Altogether, our data suggest that the present multiplex LC–MS/MS method could be used instead of the reference methods for routine TDM purpose. Five French clinical PK laboratories were involved in the cross-validation campaign, thus reinforcing the robustness of our results. As previously mentioned, we could not compare the performance of our method for Ipi. However, Ipi plasma concentrations were assayed in patients treated at different doses (i.e., 1 or 3 mg/kg) for melanoma or lung cancer. These concentrations were consistent with those previously reported elsewhere for these indications [7,49], which suggests a good reliability of our method. A cross-validation for Ipi should be conducted in the future.

Over the last decade, several mAbs (i.e., Beva, Cetux, Ritux and Trastu) have become available in most countries. Since 2018, the different health authorities worldwide have promoted the use of biosimilars in oncology as a means to cut drug costs. However, some practitioners are worried during the switch procedure from princeps mAbs to biosimilar mAb, because of concerns regarding possible loss of efficacy as PK of biosimilar may not perfectly match that of the princeps mAb. The use of TDM before and after such a switch could help to ensure that plasma mAb levels remain stable with biosimilars. In this context, a versatile LC–MS/MS method capable of assaying both originator and biosimilar mAbs would be very useful. The present multiplex LC–MS/MS method exhibited a similar performance (accuracy and precision) to assay plasma concentration with actual Herceptin^®^ and biosimilar Trastu. This result suggests the analytical reliability of our method, regardless the mAb. However, this reliability should be further confirmed with other biosimilars of Beva, Cetux and Ritux, for fully confirming that our technique enables assaying both originator and biosimilar mAbs.

Despite the relatively long run time of our assay, its versatility is a major asset when implementing routine TDM for various reasons: Samples can be gathered in a unique laboratory and results can be released more quickly. Thus, the loss of time caused by a longer run time is offset by the large benefits of multiplexing. Overall, this may help spreading the monitoring of mAbs in cancer patients as a daily clinical practice. Furthermore, the treatment paradigm of some cancers such as melanoma and lung cancer has dramatically changed in recent years with the introduction of immunotherapy. A better understanding of PK/PD relationship for ICI therapies could contribute to optimizing individual treatment in the era of personalized medicine [7,15]. 

## 4. Materials and Methods 

### 4.1. Reagents and Consumables

mAbXmise kit-multiplex 7 mAbs (Beva, Cetux, Ipi, Nivo, Pembro, Ritux, Trastu) was obtained from Promise Proteomics (Grenoble, France). mAbXmise is a ready-to-use kit enabling the simultaneous plasma quantification of 7 mAbs using full-length isotopic versions of each as internal standards (SIL–Trastu, SIL–Beva, SIL–Cetux, SIL–Ritux, SIL–Nivo, SIL–Ipi, SIL–Pembro). mAbXmise kit contains all reagents, calibration standards (*n* = 6 including zero), three levels of IQCs (2 IQCs included in the kit and 1 additional IQC made for the validation), consumables (mAbXmise plate, PuriXmise plate) and solutions (CutXmise enzyme and CutXStop) to prepare samples before the LC–MS/MS injection. LC–MS/MS grade acetonitrile and formic acid were purchased from Merck-Sigma (St. Louis, MO, USA) and Fisher Chemicals (Illkirch, France), respectively. Ultrapure water (resistivity 18.2 mΩ cm) was obtained using a Milli-Q Plus^®^ system (Millipore, Molsheim, France).

### 4.2. Calibration Curve and Internal Quality Control Preparation

Calibration curves and IQCs were designed according to the expected concentrations for the 7 mAbs. Seven independent stock solutions were prepared for each mAb: one was used for the preparation of calibration standards and the other for the IQCs. All stock solutions were prepared from reference solutions of Trastu (Herceptin^®^, 120 mg/mL), Beva (Avastin^®^, 25 mg/mL), Cetux (Erbitux^®^, 5 mg/mL), Ritux (Mabthera^®^, 10 mg/mL), Nivo (Opdivo^®^, 10 mg/mL), Ipi (Yervoy^®^, 5 mg/mL) and Pembro (Keytruda^®^, 25 mg/mL) obtained from Myonex (Leicester, United Kingdom). Briefly, a volume of 4 mL of pre-diluted CAL and IQC solutions, at 10, 50, 125, 250, 500, 30, 75 and 375 µg/mL, were prepared in PBS 1X. Then, the 4 mL pre-diluted solutions were diluted in 16 mL of blank plasma to get the calibration standards and IQCs at the final concentrations: 2, 10, 25, 50, 100 and 6, 15 and 75 µg/mL.

#### 4.2.1. Sample Preparation with mAbXmise Kit

**Figure 5:**
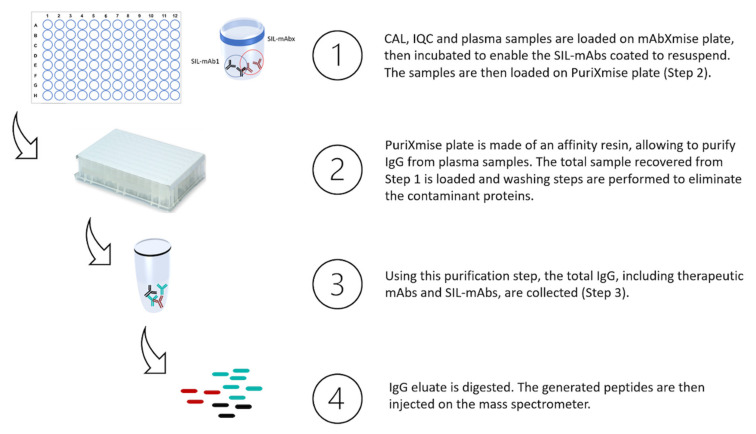
mAbXmise process summarized: collected plasma samples are loaded on mAbXmise plate as well as calibrators and QC samples provided in the kit. Full-length isotopically labelled mAbs, coated on the plate, are resuspended in the plasma samples and will serve as internal quantification standard. Total IgG are purified, recovered, and then digested. At the end of the process, the samples collected are ready to be injected.

Samples were prepared according to manufacturer’s instructions. Briefly, samples were prepared as follows (Figure 5): 20 µL of plasma sample (calibration standard, IQC or patients’ sample) were loaded in wells of the mAbXmise plate and diluted with 80 µL of Buffer A solution provided in the kit. Then, they were agitated for 1 h at room temperature. The 7 mAbs as well as their full–length isotopically labelled forms (SIL–mAbs) were extracted by immunocapture on the PuriXmise plate. After an elution step, extracted samples were dried in a speed vacuum (Martin Christ, Osterode am Harz, Germany). Samples were re-solubilized and then digested with CutXmise enzyme overnight at 37 °C. Digestion was stopped with CutXStop, then 20 µL of digested samples were injected in the LC–MS/MS system.

#### 4.2.2. Selection of Peptides for Quantification

The final list of proteotypic peptides selected and their corresponding MRM transitions is given in Table 3. For all listed peptides, the MRM transitions were used as “quantifier” as all gave consistent quantification data. In the presented data, the mean of multiple MRM transitions was used to calculate mAb final concentration.

#### 4.2.3. Chromatographic and Mass Spectrometric Conditions and Instrumentation

The chromatographic system used consisted of an Exion system with binary pumps, autosampler set at 15 °C, and a column oven maintained at 40 °C (Sciex, Framingham, MA, USA). Chromatographic separation of peptides was achieved using a BioZen™ 2.6 µm Peptide XB–C18 LC column 100 × 2.1 mm (Phenomenex, Torrance, CA, USA) or a XSelect CSH C18 LC column 100 × 2.1 (Waters, Milford, MA USA). A gradient elution program was conducted for chromatographic separation with mobile phase A (water, 0.1% formic acid) and mobile phase B (acetonitrile, 0.1% formic acid) as follows: 0–1 min (95% A), 1–2 min (from 95% to 80% A), 2–12 min (from 80% to 60% A), 12–12.1 min (from 60% to 10% A), 12.1–14.5 min (10% A), 14.5–14.6 min (from 10% to 95% A), 14.6–20 min (95% A). The flow rate was 100 µL/min. Analysis for method validation was performed using a 6500 QTRAP (Sciex, Framingham, MA, USA). Source conditions were optimized with the curtain gas at 20 psi, Ionspray voltage at 5500 V, temperature at 500 °C, ion source gas 1 at 40 psi and ion source gas 2 at 45 psi. Declustering potential, entrance potential and collision cell exit potential values were 60, 12 and 19 for compound parameters. Analyses for cross-validation were performed using a TSQ Altis (Thermo Fisher Scientific, San Jose, CA, USA) for Beva, Ipi, Ritux, and Trastu, XEVO TQ-XS (Waters, Milford, MA USA) for Nivo, Pembro and Cetux. 

#### 4.2.4. Selectivity, Carry-Over and Matrix Effect

The selectivity was evaluated by analyzing plasma samples (*n* = 6) from naïve treatment cancer patients and carry-over by analyzing the signal intensities for peptides (from mAbs and IS) in a blank sample (mobile phase) injected just after the sample CAL5 (100 µg/mL of each mAb). To determine the matrix effect, a mix of the 7 pure mAbs was digested with CutXmise. This mix was then divided in two fractions (2 × 30 µL). One fraction was supplemented with 30 µL mobile phase A, while the second fraction was supplemented with 30 µL digested blank plasma from a pool of human plasma samples (*n* = 6). The peak areas of each peptide were determined in both conditions and the matrix effect for each mAb was determined by dividing the peak area in the presence of plasma by the peak area in absence of plasma.

#### 4.2.5. Method Validation 

The method was fully validated according to the EMA Guidelines for Industrial Bioanalytical Method Validation [47] for linearity, accuracy, carry-over, dilution integrity, matrix effect and selectivity. For the linearity assessment, double blank, zero samples and CAL samples (between 2 µg/mL and 100 µg/mL) were prepared and analyzed on 6 different days. Samples were prepared by spiking different known concentrations of the pure mAb solution in drug-free plasma samples as described before. The response for each mAbs was evaluated with respect to the theoretical concentration of each calibration standard. Linear regression (1/x) was applied to fit the calibration curves (area peak ratio vs. concentration). The five calibration levels in each run should be within ±15% of the nominal value, except the LLOQ which must be between ±20% of the nominal value. The regression coefficient was calculated for each analytical run and should be over 0.99. These tests were replicated six times as independent experiments. Inter-accuracy and precision were determined as four separate validation runs by injecting IQC samples (*n* = 4) at low (6 µg/mL), medium (15 µg/mL) and high concentrations (75 µg/mL) and LLOQ samples (2 µg/mL). For intra-run tests, six replicates of IQC and LLOQ samples were injected in the same day. Intra-run and inter-run accuracies were expressed as the relative bias. The intra-run and inter-run precisions were calculated as the coefficient of variation (CV). At each concentration level of IQC, the bias should be within ±15% and the precision <15%. For the LLOQ, both concentration bias and precision should be within ±20%. The instrument carry-over was tested by injecting three blank samples after an ULOQ sample. The carry-over was calculated as the ratio of the peak area in the blanks and the peak area of the LLOQ. The carry-over was considered acceptable if signal at the analyte was <20% of the LLOQ in each blank. Dilution integrity was demonstrated by diluting a plasma sample (at the concentration of 2.5 times higher than the ULOQ of each mAb) with free-drug plasma or PBS 1X by 5–fold. Six aliquots of both dilutions were processed. Both accuracy and precision should be within ±15% of the nominal value.

#### 4.2.6. Cross Validation 

All patients were treated with mAbs for solid cancers, except for Ritux. Patients receiving Ritux were treated for vasculitis. All whole blood sampling were collected as part of clinical trials. All patients provided written informed consent for blood sampling. Whole blood was collected in heparin lithium-containing tubes just prior to the next infusion (trough concentration) or at peak (end of the infusion). After centrifugation at 3000 rpm during 15 min, plasma was aliquoted in polypropylene tubes, then stored at −20 °C until analysis.

A cross validation between the multiplex LC–MS/MS method and a published reference method was conducted for all mAbs except for Ipi. The multiplex LC–MS/MS method was applied in French laboratories of pharmacology: Cochin Hospital (Paris, France) for Ipi, Beva, Ritux and Trastu; La Timone University Hospital (Marseille, France) for Nivo, Pembro and Cetux. Reference techniques [26,27,28,29,30,31] including Beva, Cetux, Nivo, Pembro, Ritux and Trastu were performed in other French laboratories of Pharmacology at Grenoble, Lyon, and Tours according to protocol recommendations previously published. All participating laboratories are GPCO-Unicancer members.

#### 4.2.7. Statistical Analysis 

All statistical analyses were performed using MedCalc statistical package version 19.2.6 (MedCalc Software, Mariakerke, Belgium). The Passing-Bablok regression was used to estimate the relationship between the multiplex LC–MS/MS method and the reference method [50]. The regression equation was expressed with the 95% confidence interval (95% CI) for the estimates of slope and intercept. The Bland–Altman plot was used to evaluate method agreement [54]. The numerical results were reported both mean bias and the limits of agreement, including respective 95% confidence intervals (95% LOA). 

#### 4.2.8. Ethic Committee Approval

The study was conducted according to the guidelines of the Declaration of Helsinki and approved by Ethics Committee: Minister or Research and Innovation (number DC2016–2739) for bevacizumab, Sud–Méditerrannée III (number 2012.03.02) for cetuximab, Sud–Est IV (number DC-2008-72) for ipilimumab, CLEC (number 2442) for nivolumab and CPP Île de France (MAINRITSAN2 study, ClinicalTrials.gov NCT02119559) for rituximab. The collection of blood samples during a regular medical visit was approved by the local review board of Oncology (Assistance Publique des Hôpitaux de Paris) for patients treated with pembrolizumab or trastuzumab.

## 5. Conclusions

We described here a completely validated multiplexed MS method for seven mAbs quantification. To our best knowledge, this work is the first to present a method that has been cross-validated in several laboratories. Moreover, it is the first approach to allow simultaneous determination of immune checkpoint inhibitors (ipilimumab, nivolumab, pembrolizumab). 

## Figures and Tables

**Table 1:** Intra- and inter-assay accuracy and precision of OTDM1 monoclonal antibodies measured in plasma with mAbXmise kit. Results obtained with originator drugs (linear regression 1/X) *n* = 3 (IQC: internal quality control; LLOQ: lower limit of quantification) ^a^ Precision is expressed as coefficient of variation (%). * *n* = 4, ** *n* = 8.

		Within-Run			Between-Run		
Nominal Concentration (µg/mL)	Level	Mean Calculated Concentration (µg/mL)	Intra-Day Precision ^a^ (%, *n* = 6)	Intra-Day Accuracy (%, *n* = 6)	Mean Calculated Concentration (µg/mL)	Inter-Day Precision ^a^ (%, D = 4, *n* = 16)	Inter-Day Accuracy (%, D = 4, *n* = 16)
BevacizumabFTFSLDTSK							
2	LLOQ	2.1	3.4	103.0	2.0	1.5	101.2
6	Low IQC	6.3	3.1	105.1	6.2	3.3	102.8
15	Mid IQC	15.5	1.8	103.1	15.8	1.7	105.4
75	High IQC	75.4	1.1	100.5	75.7	1.0	100.9
Cetuximab YASESGIPSR							
2	LLOQ	2.0	1.4	102.0	2.0	4.6	98.6
6	Low IQC	6.2	1.9	104.0	6.1	3.4	102.5
15	Mid IQC	15.2	1.9	101.0	15.4	1.7	102.4
75	High IQC	73.8	0.9	98.4	74.1	1.1	98.8
Ipilimumab GLEWVTFISYDGNNK							
2	LLOQ	2.2	3.4	111.1 *	2.0	11.5	99.4 **
6	Low IQC	6.2	14.6	102.7	6.0	7.2	100.0 **
15	Mid IQC	14.5	12.2	97.0	14.4	2.4	96.0 **
75	High IQC	81.3	3.9	108.0	78.7	5.2	105.0 **
Nivolumab ASGITFSNSGMHWVR							
2	LLOQ	2.1	6.5	103.0	2.0	12.4	100.1
6	Low IQC	5.4	6.5	90.1	5.79	13.1	96.5
15	Mid IQC	14.1	7.7	94.0	15.6	7.3	104.0
75	High IQC	74.2	2.8	99.0	74.2	1.2	99.0
Pembrolizumab DLPLTFGGGTK							
2	LLOQ	1.9	4.2	95.7	1.9	2.1	93.0
6	Low IQC	6.0	2.1	100.4	6.1	4.4	100.7
15	Mid IQC	14.9	1.4	99.3	15.4	2.5	102.3
75	High IQC	73.1	1.5	97.5	74.8	1.6	100.0
Rituximab FSGSGSGTSYSLTISR							
2	LLOQ	2.0	6.9	100.5	2.1	4.6	106.8
6	Low IQC	6.5	5.4	107.8	6.4	2.1	106.0
15	Mid IQC	16.1	3.8	107.5	16.1	1.8	107.1
75	High IQC	77.5	4.0	103.3	77.5	1.8	103.3
Trastuzumab DTYIHWVR							
2	LLOQ	2.0	7.6	98.9	2.1	5.2	103.4
6	Low IQC	5.7	3.8	95.4	6.1	5.9	101.4
15	Mid IQC	14.9	5.1	99.2	15.5	4.2	103.2
75	High IQC	72.4	2.7	96.5	74.0	1.7	98.7
Trastuzumab FTISADTSK							
2	LLOQ	1.9	3.1	93.4	1.8	8.1	91.3
6	Low IQC	6.2	2.0	103.1	6.0	4.2	99.6
15	Mid IQC	15.0	1.1	100.0	14.9	2.3	99.6
75	High IQC	74.8	1.0	99.7	73.3	1.9	97.8

**Table 2:** Summary of method agreement for each monoclonal antibody (mAb): Estimated parameters and 95% confidence interval (95% CI) of slope and intercept for each monoclonal antibody comparison. *p*-Value of Cusum test. Mean and 95% limits of agreement (LOA) of bias for each mAb comparison.

	Passing-Bablok			Bland–Altman Absolute Differences
Parameters	Slope (95% CI)	Intercept (95% CI)	Cusum Test (*p*-Value)	Bias (95% LOA)
**Bevacizumab** LC–MS/MS vs. ELISA Reference Method [26] (*n* = 16)	1.132 (1.043; 1.242)	4.16 (−1.41; 10.35)	0.58	16.1 (−8.7; 40.8)
**Cetuximab** LC–MS/MS vs. LC–MS/MS Reference Method [27] (*n* = 21)	0.829 (0.788; 0.900)	−2.72 (−4.53; 6.20)	0.72	−17.9 (−60.9; 25.0)
**Nivolumab** LC–MS/MS vs. LC–MS/MS Reference Method [28] (*n* = 21)	0.970 (0.811; 1.221)	2.59 (−3.05; 7.37)	0.72	2.7 (−7.2; 12.6)
**Pembrolizumab** LS–MS/MS vs. LC–MS/MS Reference Method [29] (*n* = 21)	1.049 (0.947;1.159)	−0.19 (−2.34; 2.04)	0.72	1.1 (−5.8; 7.9)
**Rituximab** LS–MS/MS vs. LC–MS/MS Reference Method [30] (*n* = 28)	0.909 (0.784; 1.054)	−1.30 (−5.82; 1.92)	0.89	−7.5 (−40.5; 25.5)
**Trastuzumab** LS–MS/M/S vs. LC–MS/MS Reference Method [31] (*n* = 23)	0.884 (0.740; 1.178)	19.42 (−0.23; 32.47)	0.78	13.8 (−47.8; 75.3)

**Table 3:** Surrogate peptides used for OTDM1 monoclonal antibodies quantification in plasma and their corresponding MRM transitions. All peptide listed were used as “quantifier”.

Monoclonal Antibody	Peptide Sequence	Q1 *m*/*z*	Charge Parent Ion	Q3 *m*/*z*	Fragment Ion
Bevacizumab	FTFSLDTSK	523.263664	2+	797.404	y_7_^+^
				650.336	y_6_^+^
				563.304	y_5_^+^
				450.219	y_4_^+^
				335.193	y_3_^+^
	FTFSLDTS [13C6,15N2] K	527.270764		805.418	y_7_^+^
				658.350	y_6_^+^
				571.318	y_5_^+^
				458.234	y_4_^+^
				343.207	y_3_^+^
Cetuximab	YASESISGIPSR	633.819866	2+	1103.569	y_11_^+^
				1032.532	y_10_^+^
				816.457393	y_8_^+^
	YASESISGIPS [13C6,15N4] R	638.824001		1113.577	y_11_^+^
				1042.540	y_10_^+^
				826.466	y_8_^+^
Ipilimumab	GLEWVTFISYDGNNK	871.922851	2+	1257.611	y_11_^+^
				1158.543	y_10_^+^
				1057.495	y_9_^+^
				910.426	y_8_^+^
				797.342	y_7_^+^
				710.310	y_6_^+^
				547.247	y_5_^+^
	GLEWVTFISYDGNN [13C6,15N2] K	875.929951		1265.625	y_11_^+^
				1166.557	y_10_^+^
				1065.509	y_9_^+^
				918.441	y_8_^+^
				805.357	y_7_^+^
				718.325	y_6_^+^
				555.261	y_5_^+^
Nivolumab	ASGITFSNSGMHWVR	550.599946	3+	1073.495	y_9_^+^
				986.462	y_8_^+^
				872.420	y_7_^+^
				785.387	y_6_^+^
				661.309	y_11_^2+^
	ASGITFSNSGMHWV [13C6,15N4] R	553.936036		1083.503	y_9_^+^
				996.471	y_8_^+^
				882.428	y_7_^+^
				795.396	y_6_^+^
				666.313	y_11_^2+^
Pembrolizumab	DLPLTFGGGTK	553.298038	2+	877.478	y_9_^+^
				780.425	y_8_^+^
				566.293	y_6_^+^
				419.225	y_5_^+^
	DLPLTFGGGT [13C6,15N2] K	557.305138		885.4920	y_9_^+^
				788.439	y_8_^+^
				574.307	y_6_^+^
				427.239	y_5_^+^
Rituximab	FSGSGSGTSYSLTISR	803.889009	2+	1084.563	y_10_^+^
				1027.542	y_9_^+^
				926.494	y_8_^+^
				839.462	y_7_^+^
	FSGSGSGTSYSLTIS [13C6,15N4] R	808.893143		1094.572	y_10_^+^
				1037.550	y_9_^+^
				936.502	y_8_^+^
				849.470	y_7_^+^
Trastuzumab	DTYIHWVR	545.27744	2+	873.473	y_6_^+^
				710.410	y_5_^+^
				597.326	y_4_^+^
				460.267	y_3_^+^
				630.288	b_5_^+^
				816.368	b_6_^+^
	DTYIHWV [13C6,15N4] R	550.281575		883.481	y_6_^+^
				720.418	y_5_^+^
				607.334	y_4_^+^
				470.275	y_3_^+^
				630.288	b_5_^+^
				816.368	b_6_^+^
	FTISADTSK	485.248014	2+	822.420	y_8_^+^
				608.289	y_6_^+^
				521.257	y_5_^+^
				450.219	y_4_^+^
	FTISADTS [13C6,15N2] K	489.255114		830.435	y_8_^+^
				616.303	y_6_^+^
				529.271	y_5_^+^
				458.234	y_4_^+^

## Supplementary Materials

The Supplementary Materials are available online at https://www.mdpi.com/article/10.3390/ph14080796/s1.
