# Supplementary material for: Cross-Validation of a Multiplex LC-MS/MS Method for Assaying mAbs Plasma Levels in Patients with Cancer: A GPCO-UNICANCER Study
Source: Pharmaceuticals (Basel). 2021 Aug 12;14(8):796. doi: 10.3390/ph14080796 (PMC8401780; doi:10.3390/ph14080796)
Supplement: Supplementary file 1 [file pharmaceuticals-14-00796-s001.zip › pharmaceuticals-1274520-supplementary.pdf]

Supplementary Materials

Table S1.. Accurancy and precision of Trastuzumab measured in plasma with mAbXmise kit. Results obtained with biosimilar of Trastuzumab (Ontruzant®) using two distinct peptides. [a]Precision is expressed as coefficient of variation (%).

**Trastuzumab DTYIHWVR**	**Nominal concentration (µg/mL)**	**Mean calculated concentration (µg/mL)**	**Precision[a] (%, n=6))**	**Accuracy (%) n=6**
**Ontruzant®**				
	6	6.3	8.9	104.7
	15	16.0	8.6	106.6
	75	79.7	4.0	106.3
**Trastuzumab FTISADTSK**	**Nominal concentration (µg/mL)**	**Mean calculated concentration (µg/mL)**	**Precision[a] (%, n=6))**	**Accuracy (%) n=6**
**Ontruzant®**				
	6	5.9	2.1	99.8
	15	16.3	2.2	108.7
	75	82.8	2.7	110.4

Table S2. Intra-assay accuracy and precision of OTDM1 monoclonal antibodies measured in plasma spiked at 250 µg/mL and then diluted by 5in PBS 1X or plasma, with mAbXmise kit. Results obtained with originators drugs. [a] Precision is expressed as coefficient of variation.

			Within-run	
	**Nominal concentration (µg/mL)**	Mean Calculated concentration (µg/mL)	**Intra-day precision[a] (%, n = 6)**	**Intra-day accuracy (%, n = 6)**
**Bevacizumab FTFSLDTSK**				
PBS	250	253.9	1.5	101.6
Plasma	250	257.8	1.7	103.1
**Cetuximab YASESGIPSR**				
PBS	250	259.8	2.8	103.9
Plasma	250	256.3	2.3	102.5
**Ipilimumab GLEWVTFISYDGNNK**				
PBS	250	284.9	9.32	114.0
Plasma	250	234.2	8.91	93.7
**Nivolumab ASGITFSNSGMHWVR**				
PBS	250	230.9	2.9	92.4
Plasma	250	249.2	5.9	99.7
**Pembrolizumab DLPLTFGGGTK**				
PBS	250	257.8	1.8	103.1
Plasma	250	262.3	1.4	104.9
**Rituximab FSGSGSGTSYSLTISR**				
PBS	250	267.0	3.1	106.8

Plasma	250	263.8	6.4	105.5
**Trastuzumab DTYIHWVR**				
PBS	250	238.6	1.8	95.4
Plasma	250	259.8	4.6	103.9
**Trastuzumab FTISADTSK**				
PBS	250	242.6	2.0	97.0
Plasma	250	252.3	1.8	100.9
